# The Virtual Xenbase: transitioning an online bioinformatics resource to a private cloud

**DOI:** 10.1093/database/bau108

**Published:** 2014-11-06

**Authors:** Kamran Karimi, Peter D. Vize

**Affiliations:** Department of Biological Sciences, University of Calgary, 2500 University Dr NW, Calgary, Alberta, Canada, T2N 1N4

## Abstract

As a model organism database, Xenbase has been providing informatics and genomic data on *Xenopus (Silurana) tropicalis* and *Xenopus laevis* frogs for more than a decade. The Xenbase database contains curated, as well as community-contributed and automatically harvested literature, gene and genomic data. A GBrowse genome browser, a BLAST+ server and stock center support are available on the site. When this resource was first built, all software services and components in Xenbase ran on a single physical server, with inherent reliability, scalability and inter-dependence issues. Recent advances in networking and virtualization techniques allowed us to move Xenbase to a virtual environment, and more specifically to a private cloud. To do so we decoupled the different software services and components, such that each would run on a different virtual machine. In the process, we also upgraded many of the components. The resulting system is faster and more reliable. System maintenance is easier, as individual virtual machines can now be updated, backed up and changed independently. We are also experiencing more effective resource allocation and utilization.

**Database URL:** www.xenbase.org

## Introduction

Xenbase (www.xenbase.org) is a model organism database (MOD) dedicated to supporting research on the South African clawed frog, *Xenopus laevis*, and its close relative *Xenopus* (*Silurana*) *tropicalis*, both of which are important systems for performing biomedical research (1, 2). Our goal has been to create a comprehensive resource that researchers can use to obtain the latest relevant data necessary to interpret their experimental results and to plan new experiments. Xenbase collects and makes available scientific literature, gene and genomic data from different sources, including, but not limited to, the National Center for Biotechnology Information, Unigene and Online Mendelian Inheritance in Man (OMIM) and the Joint Genome Institute (1). Data are imported through various pipelines, mined for content with automated systems and annotated by a team of professional data curators. Approximately, 1000 individual users access Xenbase services each day.

Community contributions are also supported, and Xenbase enables *Xenopus* researchers to communicate and collaborate using personal and laboratory profiles, as well as job openings and news announcements. Xenbase offers a GBrowse genome browser (3), as well as a BLAST+ (4) server and the text-mining system Textpresso (5). A variety of software enables these processes, including DB2 and MySQL databases, WebSphere application server and Perl. The application layer is written in Java and the HTTP server is Apache. All of these components run on the RedHat Enterprise Linux (RHEL) Operating System (OS).

Xenbase’s database has been increasing in size continuously. Within the past 12 months, the compressed database has grown from about 33 GB to about 50 GB, and we expect this trend to continue. Reasons include the addition of more of the existing data types (publications, genome data, etc.) as well as new data types (morpholinos, OMIM data, etc.). We are also continuously adding new images, which has a big effect of database size. As concrete examples, the database currently contains more than 45 000 publications. It has more than 15 800 gene pages, of which 1835 have community submitted images, and 1972 have literature images. We have recently added *tropicalis* 8.0 and *laevis* 7.1 genome support, while continuing to maintain the older *tropicalis* 7.1 and 4.1, as well as *laevis* 6.0 genomes.

**Figure 1.**
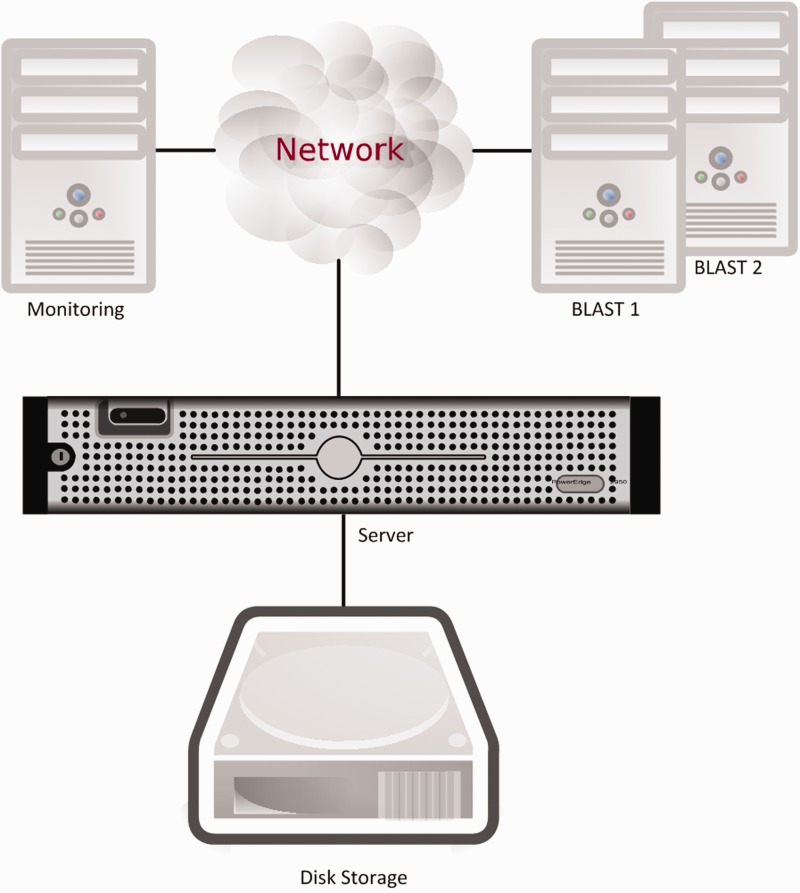
Xenbase’s original hardware setup and data transfer connections. All services except for BLAST were running on a single server.

Originally all these components were running on a single server. This had a number of inherent problems, with the major issue being reliability and availability of service. When one component failed for any reason, often the entire system was rendered unusable and the server had to be rebooted. Given the hardware speed and the number of components to load, a reboot would typically take over 20 min, an unacceptable downtime for a heavily used resource. As all the software components shared the available processors and memory, if a compute- or memory-intensive process was active other processes could be delayed or even fail. Another issue with this architecture was security, as all components were as secure, or vulnerable, as all others, and an intrusion into the system would allow access to all components. Figure 1 below shows the original setup, with one server responsible for running all services except for BLAST. System monitoring was done by running a number of scripts on a separate PC, notifying Xenbase staff if the website was not responsive.

One advantage of running all these components on a single server is the ease of integration. For example, importing and exporting data between the database and other components would only require a local copy. Data exchange was thus very fast and reliable. However, such a monolithic setup has many disadvantages. A failure in one component would often bring down the whole system. In addition, upgrading and updating the OS and the user-level components was becoming more difficult with time, as different components were evolving at different speeds. For example, the OS was changing at a much faster rate than some informatics resources. Certain components were relying on older libraries, whereas others needed newer versions. Even without the software dependency problems, maintenance work on the old setup needed careful planning. Bringing down one component to add features or fix bugs would impact the whole system’s availability.

It was clear that Xenbase’s monolithic design was approaching its limits. We needed to be able to decouple the services in order to maintain them separately in isolated environments. In this article, we describe how a new virtualized software architecture (6) helped us alleviate many of our problems.

## The Case for Virtualization

As outlined in the Introduction, we chose to decouple Xenbase’s many services and run them in independent environments. Having multiple physical servers to run the services involves a high cost in terms of space, maintenance, as well as budget. With the maturing of virtualization technologies, the best solution was to move the services to a number of independent virtual machines (VMs). Having services run on separate VMs removes space concerns and eases maintenance and resource allocation, because each VM can have its own virtual hardware, OS, monitoring and software libraries, without taking any physical space.

Another advantage of a virtual environment is ease of backing up whole systems and restoring them as needed. A VM can be exported to a file and saved in multiple locations, enabling us to restore our setup in case of catastrophic hardware and software failures. Restoring from such an exported VM backup is much faster than going through the setup and tuning process for software packages installed on a computer. The ability to take a ‘snapshot’ of a VM is another useful feature. Before an operation with uncertain outcome, one can take a snapshot of a VM. After the VM has changed, if need be, it can be restored to that snapshot, effectively undoing all the changes since the snapshot was taken.

Flexible resource allocation in a virtual environment makes it straightforward to dynamically assign the appropriate computational resources to tasks. If a VM needs more or less memory, or processors, for example, this is achieved through the virtual environment’s control interface without any physical changes. In a real-hardware setup, hardware under-equipping or over-equipping occurs frequently, resulting in either insufficient performance or wasted resources. Both situations are expensive to remedy.

A move to a virtual environment requires appropriate hardware and software resources. In a public cloud (7) hardware belongs to a third party, who creates VMs and makes them available to users. Such an environment not only removes resource management considerations from the VM owner’s work load but also allows less control and flexibility. The public cloud provider determines how much actual hardware resources each VM is allocated. As multiple VMs run on the same hardware, there is a potential for competition for resources among the VMs, and performance on a particular VM may vary depending on what other VMs are doing (8).

In order to have total control over hardware resources, one solution is using a private cloud, where all available hardware is dedicated to a single user’s VMs. In this case the user has access to all the computing resources, but instead has to manage the hardware and virtualization software. Other downsides of a private cloud include being limited to the physical resources provided by local machines, and the cost of upgrading the hardware at the end of its useful life. In other words, in a private cloud a single user has to bear the costs of hardware purchase, maintenance and upgrade, where as in a public cloud the costs, as well as the resources, are shared between multiple users.

Regardless of how a virtual environment is implemented, its promise of cost savings and increase in performance and availability made virtualization the best choice for evolving Xenbase.

## The Virtual Xenbase

We decided to implement a private cloud by utilizing two IBM x3650 M4 servers, each with 96 GB of memory, and two 2.0 GHZ 8-core hyper-threaded Intel Xeon E5-2650 CPUs, for a total of 32 hyper-threaded cores. The servers share about 10 TB of RAID 10 and RAID 5 disk storage. VMware vSphere 5.1 was chosen as the virtualization engine. The servers were installed in state-of-the-art server rooms at the University of Calgary Information Technologies (IT) department and professionally managed. VMware was set up to provide transparent VM monitoring and load sharing. The VMware installation was also managed by the staff at the University of Calgary IT department. The initial purchase costs were thus the only hardware costs incurred by Xenbase.

**Figure 2.**
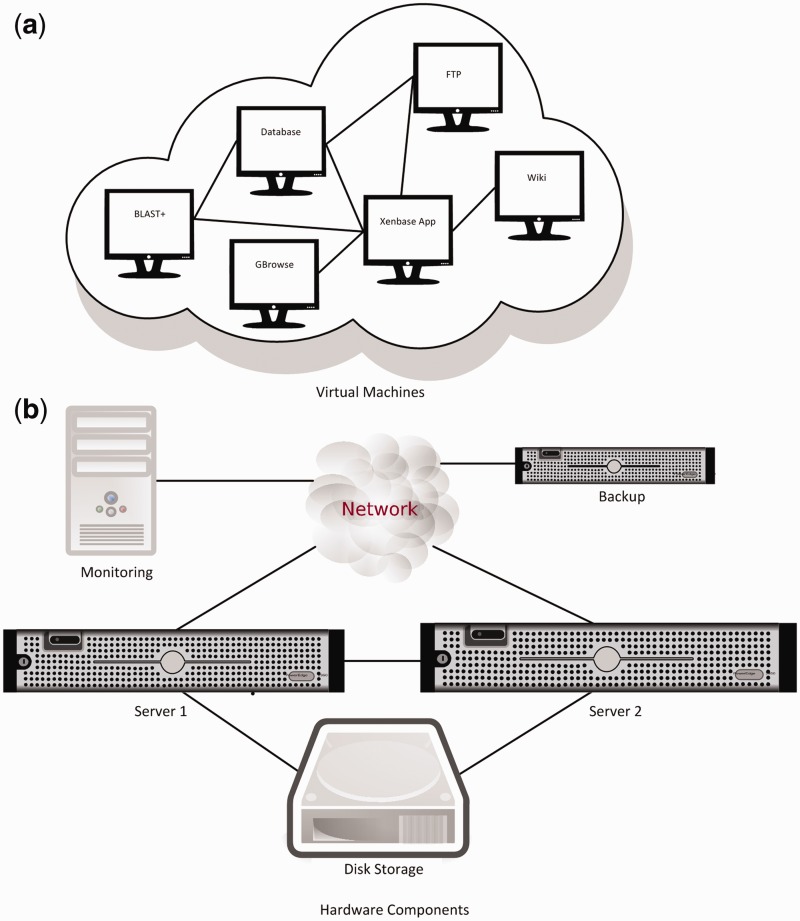
(**a**) Production VMs and data transfer paths between them. The test environment contains a dedicated application server and a GBrowse VM (not shown). (**b**) The new hardware setup and data transfer connections. VM are automatically assigned to servers and migrate between them.

We divided the major software components into a starting suite of VMs, each of which had RHEL installed as their OS. As shown in Figure 2, WebSphere Application Server, DB2 database, FTP, Wiki and GBrowse were then each installed in separate VMs. Additional software systems were then added to this base group of VMs as needed. The two BLAST machines in the old setup (Figure 1) were replaced by a single VM with 16 virtual cores and 18 GB of memory allocated to running BLAST+. At close to 1 TB, the DB2 VM is currently our biggest instance. Our smaller VM instances are around 120 GB.

Virtualization can affect performance in a negative way, but there are ways to counter that. For example, using VMware tools, we have modified all VMs’ kernels to make them aware of running in a virtual environment. This allows operations such as disk access, which are more prone to slow down in a virtual environment, to be optimized.

Figure 2 also shows Xenbase’s new physical hardware layout. The backup server uses internal disks to store data, and is located in a different building for additional protection of data. The monitoring PC is also in a different location to ensure reliable monitoring of the servers and the network. All VMs run on the two main servers. VMs can move between these servers automatically to balance load and resource usage, and also to provide fault tolerance if one of the servers malfunctions. Sharing the same external disk storage between the two main servers is required for VM migration and load balancing to be possible. Data transfer between the main servers is done through a dedicated connection to ensure maximum transfer speed independent of network load.

Apache, WebSphere and Textpresso run on the ‘Xenbase Application’ VM, which is the point of entry into the site. WebSphere and Apache are on the same VM because they both receive and respond to different visitor requests for information. Textpresso is independent of the backend database, and placing it on the same VM as WebSphere increases response time. The VMs communicate with each other through different means, including JDBC, SSH, NFS remote mounts and SFTP. In general, data transfer over the network can be slower than copying data in a single system, but this is not necessarily the situation here. If two communicating VMs are running on the same physical server, data transfer is actually done through shared memory, which is very fast compared to a real computer network. vSphere may move VMs from one physical server to another for load balancing or failure recovery, so one cannot make any assumptions about a VM’s location at any given time. If the VMs are running on separate physical systems, data transfer will be done over a dedicated connection between the servers, not the internet, so it will still be acceptably fast. As a result, system performance has not been negatively affected by the move to the new architecture. For security reasons, all VMs run their own firewalls and allow access only from trusted addresses and ports.

Upgrading BLAST to BLAST+ and using a VM with many more virtual cores and memory resulted in a very noticeable increase in BLAST performance, due to the parallel nature of the BLAST algorithm. This is the main reason we are currently operating a single BLAST+ VM. In a virtual environment adding more BLAST VMs is relatively simple, as we can clone a VM, change the necessary settings and add it to the system.

Monitoring the system is done by a number of scripts that run periodically on a separate computer, located in a different building than the main servers. Scripts typically perform a query that requires the VMs to be functioning properly to generate a correct return value. If the return does not happen within a certain time limit, the scripts inform Xenbase staff through text messages and emails. If the problem is detected outside working hours, scripts trigger a reboot of the problematic VM. This is to reduce system down time as much as possible. If a problem is detected during working hours, the affected VMs are not rebooted, allowing the staff to diagnose and solve the problem. Unlike the ∼20 min required to reboot the monolithic Xenbase system, restarting VMs typically takes <2 min.

A physical server, located in a separate building, is used to store backups of the database, as well as copies of the VMs. The production database is backed up every week, whereas entire VMs are backed up every few months to enable recovery from a catastrophic failure that may destroy the VM images. Xenbase is thus able to survive most potential problems, with a maximum loss of content equal to 7 days, and much of this can be recovered from transaction logs.

We also have a separate development and test environment, available only to Xenbase personnel. This environment contains a dedicated VM that is a copy of the production Apache/WebSphere VM, a copy of the production GBrowse VM, and a VM dedicated for biological data processing, used when generating custom datasets and reports. The DB2 VM has been allocated 64 GB of memory, and duplicating it for the test environment would reduce the memory available for other VMs. For this reason, two copies of the production instance of the database, hosted in the same DB2 VM, are used for development and testing purposes. These copies are regularly replaced with the most current version of the production database. This separate development and testing environment allows us to develop and test bug fixes and new features in an environment very similar to production, with no disruption for researchers using the Xenbase production VMs.

## Conclusions and Future Work

Concerns for scalability, reliability, performance and maintenance caused us to move Xenbase to a virtual environment. All Xenbase resources and services are running on VMs, making it one of the first MODs to do so. The purchase of VMware vSphere was the only additional software cost when moving to a virtual environment. The main cost of the move was the time and effort spent in the redesign of the system and establishing connections between the decoupled services. This has increased the complexity of the system, but the benefits of virtualization, including more flexibility and robustness, have outweighed the costs. We intend to continue exploiting this and other related technologies to improve system performance and user experience.

Our hardware is handling all current loads well, but we are processing and storing more data every day. Both the volume and the complexity of our data are increasing, and at some point our hardware resources will be insufficient for handling Xenbase’s processing and storage demands. Once this point is reached we need to determine whether to move to a public cloud, as Wormbase has recently done by moving many of its components to a cloud (9), or to upgrade our hardware. The benefits of moving to a public cloud, such as no initial hardware cost, and no hardware management and maintenance expenses, must be weighed against the advantages of a private cloud, such as dedicated hardware with predictable performance, as well as immediate access to the hardware and the infrastructure in case of problems.
